# Key Developments in the Potential of Curcumin for the Treatment of Peripheral Neuropathies

**DOI:** 10.3390/antiox9100950

**Published:** 2020-10-02

**Authors:** Martial Caillaud, Yu Par Aung Myo, Bryan D. McKiver, Urszula Osinska Warncke, Danielle Thompson, Jared Mann, Egidio Del Fabbro, Alexis Desmoulière, Fabrice Billet, M. Imad Damaj

**Affiliations:** 1 Department of Pharmacology and Toxicology, Medical College of Virginia Campus, Virginia Commonwealth University, Richmond, VA 23298, USA; myoypa@mymail.vcu.edu (Y.P.A.M.); mckiverbd@vcu.edu (B.D.M.); warnckeuo@mymail.vcu.edu (U.O.W.); thompsondc3@vcu.edu (D.T.); mannja@mymail.vcu.edu (J.M.); 2 Division of Hematology/Oncology and Palliative Care, Massey Cancer Center, Virginia Commonwealth University, Richmond, VA 23298, USA; egidio.delfabbro@vcuhealth.org; 3 Translational Research Initiative for Pain and Neuropathy at VCU, Virginia Commonwealth University, Richmond, VA 23298, USA; 4 Myelin Maintenance and Peripheral Neuropathies EA6309, Faculties of Medicine and Pharmacy, University of Limoges, F-87000 Limoges, France; alexis.desmouliere@unilim.fr (A.D.); Fabrice.Billet@unilim.fr (F.B.)

**Keywords:** curcumin, peripheral neuropathy, antioxidant, anti-inflammatory, anti-ER-stress, clinical trial

## Abstract

Peripheral neuropathies (PN) can be triggered after metabolic diseases, traumatic peripheral nerve injury, genetic mutations, toxic substances, and/or inflammation. PN is a major clinical problem, affecting many patients and with few effective therapeutics. Recently, interest in natural dietary compounds, such as polyphenols, in human health has led to a great deal of research, especially in PN. Curcumin is a polyphenol extracted from the root of Curcuma longa. This molecule has long been used in Asian medicine for its anti-inflammatory, antibacterial, and antioxidant properties. However, like numerous polyphenols, curcumin has a very low bioavailability and a very fast metabolism. This review addresses multiple aspects of curcumin in PN, including bioavailability issues, new formulations, observations in animal behavioral tests, electrophysiological, histological, and molecular aspects, and clinical trials published to date. The, review covers in vitro and in vivo studies, with a special focus on the molecular mechanisms of curcumin (anti-inflammatory, antioxidant, anti-endoplasmic reticulum stress (anti-ER-stress), neuroprotection, and glial protection). This review provides for the first time an overview of curcumin in the treatment of PN. Finally, because PN are associated with numerous pathologies (e.g., cancers, diabetes, addiction, inflammatory disease...), this review is likely to interest a large audience.

## 1. Introduction

Peripheral neuropathies (PN) can be inherited or acquired as a result of a pathological process or trauma [1]. Causes of acquired PN can have multiple origins such as autoimmune, toxic, alcoholic, diabetic, cancerous, chemo-induced, and traumatic, including crushing, constriction, stretching, and complete nerve sectioning [2,3,4]. However, the precise ethology of some neuropathies is sometimes not identified. Clinicians refer to these conditions as idiopathic neuropathies. PN may affect a single nerve (mononeuropathy), two or more nerves in different areas (multiple mononeuropathy), or many nerves (polyneuropathy). Carpal tunnel syndrome and facial paralysis are examples of mononeuropathy. Most people with PN have polyneuropathy affecting longer nerve fibers (length-dependent polyneuropathy) [5].

The symptomatology is very diverse depending on the type of nerve fibers affected. In the case of sensory fiber damage, symptoms are dependent on the caliber and size of the nerve fibers. These symptoms are generally progressive numbness, tingling in the feet or hands which may extend to the legs and arms, or even sharp, throbbing, icy or burning pain, stinging sensations and extreme sensitivity to touch. In the case of motor fiber damage, the most common symptoms are a lack of motor coordination and falls, muscle weakness or paralysis. If autonomic nerves are affected, signs and symptoms may include: heat intolerance and alterations in sweating, dermal problems, intestinal, bladder or digestive problems, but also changes in blood pressure, which can lead to dizziness [1,2].

The major clinical problem in PN, in addition to the difficulties in diagnosing and understanding pathological mechanisms, is that they are poorly treated with currently available therapeutics.

Recently, interest in the role of dietary antioxidants, such as polyphenols, in human health has led to a great deal of research on their potential as possible treatment of many inflammatory diseases. Among these polyphenols, curcumin which has long been used in traditional Asian cuisine and medicine, is an attractive molecule. However, curcumin has a very low bioavailability and a very fast metabolism. Thus, very high doses are required to achieve therapeutic effects given the uncertainty that curcumin will reach the target organ.

Curcumin [1,7-bis(4-hydroxy-3-methoxyphenyl)-1,6-heptadiene-3,5-dione], also known as diferuloylmethane, is a polyphenol present in the rhizome of Curcuma longa [6]. Curcuma longa powder is a spice known as turmeric and used in the preparation of curry. Turmeric powder is an orange-yellow crystalline compound that is used as a food coloring agent [6]. Traditionally, turmeric powder has been used in Asian countries as a medicinal preparation to combat several diseases because of its antioxidant [7], anti-inflammatory [6], antimicrobial [8], anticancer [9], and neuroprotective [10] properties. Over the past 50 years, it has been shown that most of the effects of Curcuma longa are primarily due to curcumin, with potential beneficial properties against diabetes, allergies, arthritis, neuropathies, and other chronic diseases [11]. However, there are other components of Curcuma longa that, together with curcumin, form the curcuminoid group [12]. These are demethoxycurcumin and bis-demethoxycurcumin. The curcuminoid group constitutes about 5% of the total components of Curcuma longa and curcumin is the most abundant of this group with 77% [12].

This review provides an update that is focused on pre-clinical and clinical studies investigating the use of curcumin in the treatment of PN and evaluating future therapeutic opportunities.

## 2. Curcumin Solubility, Kinetics, Bioavailability, and Metabolism

### 2.1. Solubility and Stability

The therapeutic potential of curcumin is restricted by a low solubility in aqueous solution, chemical instability, and unfavorable pharmacokinetic properties (ADME: absorption, distribution, metabolism and excretion). Due to its lipophilic nature, curcumin is practically insoluble in aqueous solution at room temperature and neutral pH [13,14]. Therefore, the use of organic solvents (such as methanol, ethanol, acetone, or dimethyl sulfoxide) is usually required. Moreover, curcumin is relatively unstable. At both neutral and alkaline pH, it degrades quickly into various compounds including ferulic acid, feruloyl methane, vanillin, and autoxidation products (bicyclopentadione primarily) [15,16,17,18]. In addition, curcumin is also sensitive to light in both solid and solubilized forms [19].

### 2.2. Bioavailability

The low bioavailability of curcumin, which has been extensively reported in rodents and humans [20,21,22], is associated with a poor absorption, a high rate of metabolism, and a rapid excretion from the body. Studies conducted in rats after oral delivery, showed that most of the ingested curcumin is excreted in feces, which accounts for its weak bioavailability. Only small amounts are absorbed within the intestine and excreted in urine [22]. Pharmacokinetics studies conducted in rats showed that the oral bioavailability of curcumin is around 1% [23]. For instance, a maximum concentration (C_max_) in the serum of only 500 ng/mL was reported after the oral delivery of 1 g/kg curcumin [24]. In another study, the C_max_ value after oral administration of 0.5 g/kg curcumin was 60 ng/mL, whereas a maximum serum concentration of 360 ng/mL was reached after i.v. delivery of 10 mg/kg curcumin [24]. In humans, a C_max_ value of 50 ng/mL was reported after oral administration of escalating doses from 500 mg to 12 g of curcumin [25]. However, while an oral dose of up to 8–12 g/day could be taken with no adverse effects [22], most of the clinical studies reported that the absorption and bioavailability of curcumin are very low since curcumin could not be detected in the serum of the majority of subjects [22]. This, in combination with the high degree of intestinal retention and retro-enteral efflux, translates to very low curcumin levels in tissue [14]. Distribution of curcumin through the body has been studied in rats where a strong variability in tissue distribution was reported [14,26,27]; however, because of the very low levels of curcumin observed in tissues, the relevance of these observations remain difficult to evaluate.

### 2.3. Curcumin Metabolism

Many studies have been carried out in rats and humans concerning curcumin metabolism, particularly in microsome fractions of intestinal or liver tissue homogenates. These studies have shown that curcumin metabolism mainly occurred via reduction followed by conjugation. Di-, tetra, hexa-, and octahydrocurcumin are the main degradation products resulting from reduction, which mainly occurs through the action of alcohol dehydrogenase [17,20,27,28,29]. Phase two metabolism, which occurs through glucuronidation/sulfonation conjugation, rapidly conjugates curcumin and its reduced metabolites [29,30,31,32]. Consequently, the small amount of curcumin that is absorbed by the body is found in the blood as glucuronide and sulfate metabolites [33].

### 2.4. Doses and Routes of Administration in PN Models

Despite its weak pharmacokinetics profile, numerous studies have reported a beneficial effect of curcumin on experimental models of PN, including peripheral nerve injury, hereditary PN (such as Charcot-Marie-Tooth (CMT) disease), alcohol-induced, chemotherapy-induced PN (CIPN), and diabetes-induced PN (DPN). As shown in Table 1, in most of these studies, curcumin was delivered alone, preferentially through oral or intraperitoneal (i.p.) route, at dose ranging from 20 to 300 mg/kg/day and for 7 to 140 days. The use of high doses appears to be required in order to achieve systemic effect. Interestingly, when a local effect is sought, curcumin has also been shown to exert beneficial effects on peripheral nerve regeneration at very low concentrations [34]. Interestingly, in vitro studies show that curcumin at doses of 0.1 to 1 μM, stimulates the proliferation, migration, and lamellipod formation of Schwann cells, protects axons from degeneration induced by neuroinflammation, and reduces oxidative stress [34,35,36]. However, since the serum concentration after oral administration of curcumin is between 10 and 500 ng/mL [22,23,24,25], the use of newer curcumin formulations to improve and better control the bioavailability of curcumin is essential.

### 2.5. New Curcumin Formulations

Recently, chemical modifications of curcumin and the development of various types of biopolymers used as carriers were shown to improve the solubility, the bioavailability, and the pharmacokinetics profile of curcumin [37,38]. However, although these new curcumin formulations allow the use of lower doses (that are compatible with clinical application), to date, only few of these formulations have been studied in the context of PN (Table 1).

## 3. Curcumin Studies in Animal Models of PN

### 3.1. Behavioral Aspects

The neuropathy models, curcumin doses, routes of administration, and species of animals in the various studies are summarized in Table 1.

#### 3.1.1. Neuropathic Pain

The symptomatology of PN is very diverse depending notably of the ethology. Neuropathic pain and hypersensitivity are reported in chronic constriction injury, nerve ligature, brachial plexus avulsion, diabetic, alcohol, and CIPN. The tests used to measure pain-related behaviors in animal models of neuropathies are hot/cold-plate, tail-flick immersion, Hargreaves and acetone tests for cold and thermal hypersensitivity. Von Frey filaments, pin prick, and Randall-Selitto tests are, for their part, used to measure mechanical hypersensitivity. Numerous studies have shown that curcumin and its derivatives have an antinociceptive effect by decreasing mechanical, thermal, and cold hypersensitivity in PN models. For example, curcumin, using oral or i.p. injections, decreased mechanical, thermal, and cold hypersensitivity in three models of CIPN (oxaliplatin, vincristine, and cisplatin) on male Sprague Dawley (SD) rats [39], on male Wistar rats [40,41] and on male Swiss albino mice [42]. In addition, tetrahydrocurcumin, a major metabolite of curcumin (see above), significantly decreased vincristine-induced hypersensitivity in male Wistar rats, measured via hot/cold plate and Randall-Selitto tests [43]. The antinociceptive properties of curcumin and its derivatives have also been reported in models of streptozotocin (STZ)-induced DPN. Curcumin reduced mechanical, cold, and thermal hypersensitivity in male Wistar and SD rats [44,45,46,47,48] and male Laka albino mouse models of DPN obtained by injection of STZ [49]. New formulations of curcumin have also shown an antinociceptive effect on mechanical, cold, and thermal hypersensitivity in models of DPN with sometimes superior effects to unformulated curcumin. For example, Ly et al. 2018, showed in male SPF rats that curcumin J147 (hybrid molecule between curcumin and cyclohexyl-bisphenol-A) decreased STZ-induced mechanical hypersensitivity in the von Frey test [50]. In addition, an alternative curcumin delivery system, self-nano-emulsifying drug delivery system (SNEDDS) curcumin, reversed, in male SD rat model of DPN, the mechanical hypersensitivity to hot and cold measured by von Frey test and tail flick hot and cold immersions, respectively [51]. Nanoparticle-encapsulated curcumin (curcumin-polybutylcyanoacrylate nanoparticle-encapsulated particles: PEGMA-DMAEMA-MAO) has also been investigated in DPN male SD rat model and showed a reduction of both thermal and mechanical hypersensitivity [52]. Other models widely used in the study of neuropathic pain are the chronic constriction injury (CCI) and nerve ligature models. In these models, animals develop chronic inflammatory pain. Thus, in particular by its anti-inflammatory action, curcumin reduced mechanical and thermal hypersensitivity in the CCI model in SD rats [53,54,55], in Wistar rats [56], in male C57BL/6J mice [57], and in nerve ligature model in female Wistar rats [58] and in BALB/c mice [59]. Finally, curcumin also showed antinociceptive properties in SD rats in models of brachial plexus avulsion [60], alcohol-induced neuropathy in male and female Wistar rats [61,62], opioid-induced hyperalgesia in C57-BL/6J mice [63], HIV-gp120-induced neuropathic pain in male SD rats [64], and complete Freund’s adjuvant-induced inflammatory pain in Charles-Foster strain rats [65].

While the use of different doses, curcumin derivatives, rodent species and strains made comparisons between studies difficult, the results show clearly that curcumin possesses antinociceptive properties in various animal models of PN. However, the majority of these studies used evoked responses (reflexive tests) as pain-related measures and no spontaneous pain assessments were reported.

#### 3.1.2. Loss of Sensitivity

A number of PN do not present pain but, on the contrary, loss of sensitivity. This is particularly the case in pathologies of genetic origin (CMT for example) or of traumatic origin (nerve crushing and transection). In this case, the substantial loss of sensory nerve fibers leads to loss of sensitivity. This loss or decrease in sensitivity can be measured in animal models by the same tests as described above. For example, curcumin, because of its neuro-protective effects, has shown to improve mechanical and thermal sensitivity in models of sciatic nerve crushing. In a first study, curcumin was administered locally as close as possible to the lesion site (with an osmotic mini pump) and showed a faster recovery of mechanical sensitivity as measured by the von Frey test in male SD rats [34]. A second study, also conducted in a male SD rat sciatic nerve crush model, showed a faster recovery of mechanical and thermal sensitivity after i.p. injection of curcumin and was dose-dependent [66].

#### 3.1.3. Motor Dysfunctions

Finally, if the motor nerve fibers are damaged (which is the case in many neuropathies), there is a loss of contact with the muscle fibers and thus motor dysfunctions. Motor behavior is thus studied in animals by various tests such as, locomotor activity by activity meter test, locomotor coordination by rotarod test, walking track analyze by sciatic function index (SFI), finger spacing by visual static sciatic index (SSI), muscular strength by grip strength test, and skilled locomotion by beam walking test. Curcumin, because of its neuro-protective effect and its ability to stimulate axonal regrowth, has shown to significantly improve motor function in various models of PN. Thus, it has been reported that curcumin improved locomotor activity in both cisplatin- and oxaliplatin-induced neuropathies in Wistar rats [40]. In addition, curcumin and tetrahydrocurcumin improved the SFI score and locomotor coordination in a vincristine-induced PN, in male swiss mouse and Wistar rat respectively [42,43]. Curcumin also allowed a faster recovery of locomotor activity in CCI model [54], of locomotor coordination in alcohol-induced neuropathy [62] and in DPN models [67]. Curcumin improved locomotor coordination, walking track analysis, finger spacing, muscular strength, and skilled locomotion in traumatic neuropathies induced by sciatic nerve crush or transection in male SD rats [34,66], in female Wistar rats [68], and in male Wistar rats [69]. Finally, locomotor coordination and muscle strength were significantly improved in CMT1A mouse model (*Trembler*-J mice C57BL/6J) [70,71], CMT1A rat model (PMP22, SD) [72] and CMT1B mouse model (heterozygous R98C mice) [73]. For example, in CMT1B model, overexpression of the myelin protein myelin protein zero (MPZ), which induces cytotoxicity in Schwann cells (SCs) and thus locomotor dysfunction, was reduced by the anti-ER-stress effect of curcumin [73].

### 3.2. Electrophysiology Aspects

Electrophysiological measurements are an important component in the assessment of PN. These are easily comparable measurements between animals and humans. These measurements are in the majority of cases performed on the caudal nerve of the animal’s tail or by exposing the sciatic nerve. Two main parameters are measured, nerve conduction velocity and signal amplitude. The reduction in nerve conduction velocity is closely related to a reduction in the thickness of the myelin sheath, while the reduction in signal amplitude corresponds to a reduction in the number of nerve fibers. It has been shown that because of its lipophilic character, curcumin is easily and stably inserted into the lipid bilayers of the myelin sheath and thus can have a protective effect on the myelin sheath thus improving nerve conduction [34]. Curcumin has been shown to improve the electrophysiological parameters in three CIPN models. For example, Zhang et al. 2020 showed in male SD rats that the decrease in motor nerve conduction velocity (MNCV) and sensory nerve conduction velocity (SNCV) observed after oxaliplatin injection were both increased after curcumin administration and were dose-dependent [39]. In addition, curcumin reverses the reduction of MNCV in the cisplatin model in female Wistar rats [41]. Similarly, tetrahydrocurcumin also restored MNCV in a vincristine-induced neuropathy model in male Wistar rats [43]. Curcumin has also shown beneficial effects on electrophysiological parameters in other chronic inflammatory neuropathies such as diabetes and alcohol. For example, two formulations of curcumin J147 and SNEDDS improved MNCV in models of DPN in female Swiss mice [67] and in male SD rats [51] and curcumin improved MNCV in ethanol-induced neuropathy model in male and female Wistar rats [61]. Finally, in models of traumatic and genetic neuropathies, in which nerve fibers and the myelin sheath are damaged, curcumin improved nerve conduction velocity and amplitude. Indeed, whether in models of sciatic nerve crush in female Wistar rats [68] and in male SD rats [34,66] or in models of complete nerve transection in BALB/c mice [74], curcumin allowed faster recovery of electrophysiological parameters, such as increased nerve conduction velocity and signal amplitude. Finally, compound muscle action potential amplitudes were also increased by curcumin treatment in a model of demyelinating neuropathy of genetic origin in CMT1A rats [72] and R98C mice (CMT1B) [73].

### 3.3. Histological Aspects

**Figure 1:**
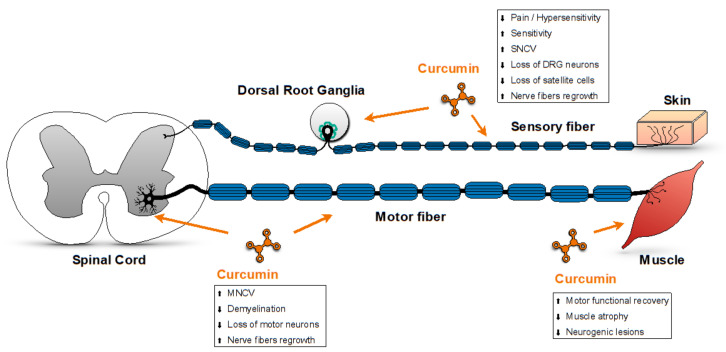
A suggested model summarizing the sites of action of curcumin in peripheral neuropathies as reported in the literature: curcumin reduces neuropathic pain and improves sensitivity in in vivo models of PN, by improving sensory nerve conduction velocity (SNCV), reducing the loss of neurons and satellite cells in dorsal roots ganglia (DRG), and promoting the regrowth of sensory nerve fibers. In addition, curcumin improves motor functions in in vivo models of PN, by improving motor nerve conduction velocity (MNCV), reducing nerve fibers demyelination, loss of motor neurons in spinal cord, muscle atrophy and neurogenic lesions, and improving motor nerves fibers regrowth.

The sites of action of curcumin in peripheral neuropathies as reported in the literature are summarized in Figure 1.

#### 3.3.1. Nerve Fibers and Myelin

PN primarily affect the nerves and notably result in a degeneration of the axons, known as Wallerian degeneration, and in a demyelination of these same axons. In some neuropathies only the axons are affected and in others only the myelin sheath. However, in the majority of cases both axons and myelin are affected. In the study published by Caillaud et al. 2018, curcumin increased the expression of myelin protein zero and myelin basic protein 22, and the thickness of the myelin sheath, and decreased neurogenic lesions of the sciatic nerve [34]. In spite of the differences in the route of administration, Ma et al. 2013 showed similar results where daily i.p. injections of 100 and 300 mg/kg of curcumin for 4 weeks promoted faster nerve regeneration after peripheral nerve injury [66]. Similar outcomes were found in a complete amputation of sciatic nerve in Balb/c mice. Intragastrical administration of 20 and 40 mg/kg/day curcumin for 1 week positively affected nerve regeneration and functional recovery in 8 weeks post-surgery. Histological analysis showed dose-dependent myelin sheath thickening with 40 mg/kg/day being the more effective in reversing damage to the nerve [74]. Rats that underwent CCI or CCI-chronic constriction release of the sciatic nerve also showed after curcumin oral treatment greater axonal regeneration and weaker degeneration of nerve tissues [75]. In hereditary neuropathy models of CMT, a 90-day curcumin treatment by oral gavage starting at postnatal day 4 in R98C CMT1B and Tremble-J mice, and i.p. injection in CMT1A rats, increased the number of large-diameter axons, higher average of fibers, axonal size, and myelin thickness in the sciatic nerve [71,72,73]. Nerve fiber degeneration and swelling, wide endoneurial space, disrupted myelin sheath, as well as swelling of SCs are histological markers of alcohol-induced neuropathy in rats. Administration of 60 mg/kg curcumin decreased these effects and also had a regenerative effect on the nerve fibers in the sciatic nerve [62]. Likewise, male rats concomitantly treated with chemotherapeutic drugs, such as cisplatin and oxaliplatin, and curcumin showed improvements in functional outcome and myelin loss by decreasing demyelination [40]. Tetrahydrocurcumin, had a dose-dependent protective action against nerve damage and axonal swelling. The higher dose of tetrahydrocurcumin (80 mg/kg) showed a better neuroprotective activity than the 40 mg/kg, with no axonal swelling of the sciatic nerve [43].

#### 3.3.2. DRGs and Spinal Cord Neurons

The dorsal root ganglia (DRG) are active participants that relay signaling from the periphery to the central nervous system [81]. Therefore, it is not a surprise to find pathological changes in these DRG in PN. For example, morphological changes in the DRG were observed in rat sciatic nerve crush model in particular on the neuronal population type A and B and satellite cells. The addition of curcumin showed a 17% type A and 36% type B cells increase compared to the untreated sciatic nerve crush group. Satellite cells (glial cells that cover the surface of DRG neurons) also showed a decrease after sciatic nerve crush and the curcumin-treated group showed a 19% increase in satellite cells compared to the untreated sciatic nerve crush group [76]. The impact of CCI in DRGs includes vacuolization, increase in the sizes of cells due to swelling, and loss of nuclei. These changes partially decreased in the curcumin-treated animals [75]. Morphometric analysis of DRGs from cisplatin-treated rats revealed a significant atrophy of the nuclei and nucleoli. When treated with curcumin (200 mg/kg/day by gavage, 5 weeks), the nucleolar atrophy was prevented, and the nuclear atrophy was partially blocked. Curcumin was also found to partially reduce the loss of DRG neurons [41]. While CIPN primary targets mainly involve peripheral nerves, recent data suggest direct toxicity in spinal cord neurons [82]. The functional state of neuronal cells can be assessed by formational changes in the Nissl bodies [83]. Spinal cord analysis of rats subjected to oxaliplatin showed curcumin-induced repairs injury in the spinal dorsal horn. Treatment with curcumin, 12.5, 25, or 50  mg/kg by gavage for 28 consecutive days, showed neatly arranged neurons that had wide intercellular space and a small number of Nissl bodies compared to the negative control. The higher dose of curcumin showed organized neurons with compact intercellular spacing and no Nissl body fragmentation [39].

#### 3.3.3. Muscles

Denervation of the gastrocnemius muscles leads to muscle neurogenic lesions seen as clusters of small skeletal muscle fibers with central nuclei. General muscle atrophy can also be reported because of a decrease in the size of muscle fibers. Caillaud et al. 2018 reported that local delivery of curcumin (0.25 μL/h) via a mini osmotic pump for five weeks post sciatic nerve crush improved the diameter of muscle fibers of rats. In addition, this study showed positive effect of curcumin on gastrocnemius muscle fibers repartition limiting the presence of clusters of small skeletal muscle fibers [34]. In another study on nerve crush injury model, rats treated with curcumin showed a motor functional recovery and reversal of gastrocnemius muscle atrophy after four weeks of daily treatment post-surgery. The recovery rate was dose-specific where groups treated with more potent curcumin solutions (100 mg/kg or 300 mg/kg) showed a significant increase in the average of muscle fiber area as compared to the vehicle and 50 mg/kg-treated group. All three doses of curcumin enhanced motoneurons regeneration which was concluded by a significantly higher number of myelinated axons per nerve transverse section and a higher mean diameter of nerve fibers than that in the vehicle group [66]. Similar results were observed by another group in a sciatic nerve crush in rats. Rodents administered with daily 100 mg/kg of curcumin (i.p.) for 4 weeks after the surgery presented smaller extent of gastrocnemius muscle atrophy, measured as muscle weight; and larger muscle diameter when compared to sham and vehicle-treated groups [77].

## 4. Curcumin Mechanisms of Action and Cellular Targets in the Treatment of PN

**Figure 2:**
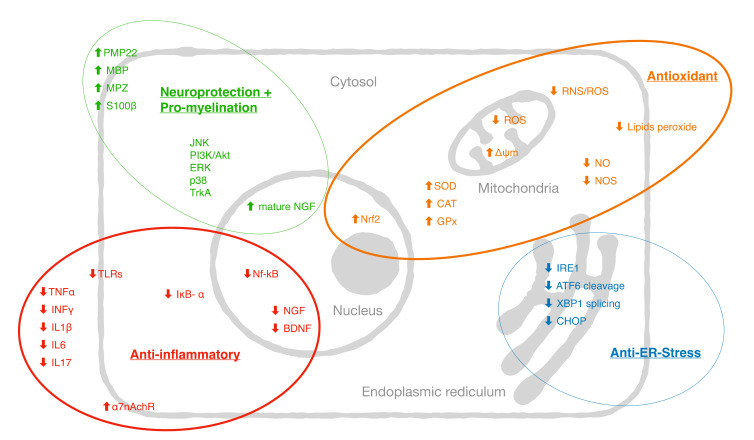
A proposed model summarizing the molecular targets of curcumin in peripheral neuropathies reported in the literature: tumor necrosis factors (TNFα), interferon (INF-γ), interleukins (IL-1α, IL-1β, IL-6, IL_10), granulocyte-macrophage colony-stimulating factor (GM-CSF), monocyte chemoattractant protein-1 (MCP-1), and macrophage inflammatory protein (MIP-1α), Toll-like receptor (TLR, TLR1, TLR3, TLR4, and TLR7), nuclear factor-kappa B (NF-κB), NF-κB inhibitor-α (IκB-α), nerve growth factor (NGF), brain derived neurotrophic factor (BDNF), lipooxygenase (LOX), cyclooxygenase (COX), nitric oxide synthase (NOS), superoxide dismutase (SOD), catalase (CAT), glutathione peroxidase (GPx), transient receptor potential cation channel subfamily-M-2 (TRMP2), nuclear factor erythroid 2–related factor 2 (Nrf2), reactive oxygen species (ROS), reactive nitrogen species (RNS), peripheral myelin protein 22 (PMP22), myelin protein zero (MPZ), myelin basic protein (MBP), extracellular signal regulated kinase (ERK), c-Jun-N-terminal kinase (JNK), α7-nicotinic acetylcholine receptors (α7-nAchR), tropomyosin receptor kinase A (TrkA), phosphoinositide 3-kinase (PI3K), activating transcription factor 3 (ATF3), ER-residing protein endoplasmic oxidoreductin-1 (Ero-1β), activating transcription factor 6 (ATF6) cleavage, X-Box binding protein 1 (XBP1) splicing, and C/EBP homologous protein (CHOP).

The molecular targets of curcumin in peripheral neuropathies reported in the literature are summarized in Figure 2.

### 4.1. Curcumin’s Anti-Inflammatory Properties

Chronic or acute inflammation is a physiological process present in all PN with a preponderance in DPN, CIPN, toxic, and traumatic neuropathies. For example, nerve damage in the peripheral nervous system has been previously established to induce Wallerian degeneration, a highly inflammatory response in which SCs that dissociate from damaged axons release cytokines such as tumor necrosis factors (TNFα), interferon (INF-γ), interleukins (IL-1α, IL-1β, IL-6, IL_10), granulocyte-macrophage colony-stimulating factor (GM-CSF), monocyte chemoattractant protein-1 (MCP-1), and macrophage inflammatory protein (MIP-1α) [84]. Upon dissociation, this cytokine release is mediated by an increase in SCs of Toll-like receptor (TLR) expression, specifically TLR1, TLR3, TLR4, and TLR7 which bind to both endogenous and exogenous ligands [85]. The downstream effect is the activation of nuclear factor-kappa B (NF-κB) [86], the release of cytokines and the recruitment of macrophages, lymphocytes, neutrophils and mast cells, which further increases inflammation [85]. The type of leukocyte recruited varies across different pathologies and mediates symptoms of PN. One of curcumin’s beneficial properties is its anti-inflammatory effects. In in vitro studies, it decreases the activation of NF-κB downstream of TLRs by decreasing the phosphorylation of its regulator, NF-κB inhibitor-α (IκB-α) [86]. Unphosphorylated IκB-α binds to NF-κB and prevents its translocation to the nucleus, thus inhibiting downstream expression and release of cytokines [86]. Similarly, in vivo studies have shown curcumin to dose-dependently downregulate the expression of INF-γ [87], TNF-α [39,43,60,61,67,87,88], IL-1β [39,61,87], IL-17 [87], IL-6 [39,61,87], and C-reactive protein [67]. This downregulation of cytokines is seen systemically in the sciatic nerve, whole brain, and spinal cord samples of autoimmune neuritis, alcoholic neuropathy, sciatic nerve injury, chemotherapy-induced peripheral neurotoxicity, and various rodent models used in the studies above. Nerve growth factor (NGF), c-Fos [60], brain-derived neurotrophic factor (BDNF) [53], and Cox-2 [53] are some markers for inflammation-mediated pain [60]. As curcumin has the capacity to reduce the inflammatory response, spinal cord expression of both NGF and c-Fos was reduced in a brachial plexus avulsion model [60]. The assumption is that the NGF measured was pro-NGF, since this is the form of NGF involved in neuronal apoptosis and death [78]. p300 and CREB-binding protein (CBP) histone acetyl transferases (HATs) mediate the expression of the other two markers, BDNF and Cox-2 [53]. Chronic constriction injury models treated with curcumin had reduced promoter acetylation and subsequent reduced expression of BDNF and Cox-2 in the spinal cord [53]. The decrease in expression paired with behavioral findings of lower threshold for mechanical, heat, and cold allodynia provides support for curcumin’s anti-nociceptive effects via its anti-inflammatory pathways [53,60].

### 4.2. Curcumin Reduces Oxidative Stress

Oxidative stress and inflammation are closely related pathophysiological processes, one of which can be easily induced by the other. Hence, when peripheral nerve damage induces an inflammatory response, oxidative stress will also be activated. Oxidative stress encompasses the production of reactive oxygen and nitrogen species (ROS/RNS) such as hydrogen peroxide, superoxide radicals, hydroxyl radicals, and nitric oxide radicals which imposes cellular damage [87]. Lipooxygenase (LOX), cyclooxygenase (COX), and nitric oxide synthase (NOS) play major roles in their production while superoxide dismutase (SOD), catalase (CAT), and glutathione peroxidase (GPx) are involved in their elimination [89]. Curcumin decreased lipid peroxide [34,39,42,43,61,62], nitrite [61], NOS [67], and NO [42,43] levels. In the optic nerve, ROS has been reported to activate transient receptor potential channels, especially transient receptor potential cation channel subfamily-M-2 (TRMP2), which causes an influx of Ca^2+^, thus mediating nerve damage [90]. TRMP2 activation was reduced with curcumin, resulting in decreased mitochondrial membrane depolarization and further decrease in the production of reactive oxygen species (ROS) and reactive nitrogen species (RNS) [90].

Antioxidative mechanisms are also upregulated by curcumin treatment. Nuclear factor erythroid 2–related factor 2 (Nrf2), a transcription factor which activates the antioxidant response element (ARE), is upregulated with curcumin [34]. The downstream effect of ARE activation is the production of antioxidative enzymes, and accordingly, curcumin was reported to increase SOD [39,42,43,86], CAT [39,42,43,86], and GPx [42,43] activity. This increase was observed in the sciatic nerve and cortical neurons of in vivo experimental rodent models of CIPN. Taken together, curcumin has the ability to: (i) Decrease the production of total and mitochondrial ROS/RNS; (ii) decrease lipoperoxidation of membrane lipids; and (iii) increase levels of antioxidant enzymes (SOD, CAT, GPx) mediated by transcription factor Nrf2.

### 4.3. Curcumin Relieves Endoplasmic Reticulum (ER) Stress

A growing body of evidence shows a strong link between oxidative stress and ER stress in neurological diseases [91]. Indeed, the ER redox environment dictates the fate of entering proteins in ER [91]. Thus, an increase in intracellular oxidative stress is unfavorable to the proper folding of proteins. Protein misfolding and ER stress have been commonly reported in PN, such as CMT disease [73,92]. CMT disease is the most common inheritable PN with various subtypes (CMT1, CMT2, DI-CMT, CMT4, and CMTX…) [2]. Of these, CMT1, especially CMT1A and CMT1B, involves demyelination of peripheral nerves [2]. CMT1A and CMT1B are caused by mutations in peripheral myelin protein 22 (PMP22) [72] and myelin protein zero (MPZ) respectively, resulting in misfolded protein accumulation in the ER of SCs [93]. This accumulation activates the unfolded protein response (UPR) which causes ER stress, oxidative stress, and subsequent apoptosis of SCs [94]. Curcumin dose-dependently decreased the accumulation of mutant proteins in the ER [92], apoptosis rate [70,92], UPR markers activating transcription factor 3 (ATF3), ER-residing protein endoplasmic oxidoreductin-1 (Ero-1β) [71], activating transcription factor 6 (ATF6) cleavage, X-Box binding protein 1 (XBP1) splicing, and C/EBP homologous protein (CHOP) [73]. The effect of curcumin on Chop expression remains unclear as no effect was observed in Okamoto et al.’s 2013 study while in Patzko Bai et al.’s 2012 study a decrease in Chop was seen that did not undergo nuclear translocation [71,73]. In addition, a limitation of these studies is the use of HeLa and HEK293 cells, very far from the SC phenotype. Nevertheless, the hypothesized mechanism of action for curcumin’s ER stress relief is proposed to be through the modulation of ER calcium levels, impairing calcium-dependent chaperones (calnexin, calreticulin) and subsequently decreasing the UPR or activation of endoplasmic-reticulum-associated protein degradation (ERAD) pathway [72,73,92]. Additionally, the expression of heat shock protein 70 (Hsp70) appears to be an important factor in curcumin’s UPR reduction [71,72].

### 4.4. Curcumin Recruits Schwann Cells, Induces Remyelination and Nerve Regeneration

Peripheral nerve demyelination develops in conditions such Guillain-Barre syndrome (GBS), chronic inflammatory demyelinating polyradiculoneuropathy (CIDP), paraneoplastic neuropathy, CMT, vitamin deficiencies, and CIPN [2]. Demyelination can be mediated through various mechanisms, from autoimmunity (CIDP) to SC dissociation from axons, SC apoptosis, oxidative stress, and inflammation [95]. Myelination by SCs is mediated by the NF-κB pathway, wherein axons and laminin induce the phosphorylation of p65 which activates NF-κB, a pro-myelinating transcription factor as seen above [35,73]. Curcumin induces remyelination by increasing NF-κB levels [35] and by increasing MPZ [34], PMP22 [34,79], S100β [69,74,77,79], and MBP levels [35,79]. These markers are indicative of increased SC recruitment and promyelinating activity. As such, an increase in myelin thickness [34,40,41,66,69,70,76,80] and axon diameter [34,66,70,74,76,80] is observed with curcumin treatment. Also, of note is the proposed involvement of extracellular signal-regulated kinase (ERK) and p38 kinases [35]. The former promotes myelination and the latter the opposite. Accordingly, treatment with curcumin yielded an increase in ERK and a decrease in phosphorylated p38 [35] in SC cultures, providing more evidence for its remyelination properties. Demyelination upon insult is typically followed by axonal degeneration and this can be mediated by the activation of the microglial TLR-4/My88 and axonal c-Jun-N-terminal kinase (JNK) pathways [36]. Curcumin interferes with the JNK pathway in axons to exert its neuroprotective effects [36,66,69], in both in vitro (hippocampal neurons) and in vivo models of sciatic nerve transection and sciatic nerve crush injury. Similarly, curcumin downregulates JNK and upregulates promyelinating Krox-20 expression in SCs in CMT 1B model [73]. In PN such as CIPN, the effects are not limited to the extremities and can affect the DRG. This is reflected in the reduction in neuronal size/volume and the number of DRG neuronal population [41,76]. Curcumin’s effects are systematic as they increased DRG neuron size and population in CIPN and in sciatic nerve crush models [41,76], with the proposed mechanism being via curcumin’s antioxidative properties [41]. Some other ways of assessing nerve regeneration are by looking at nerve fibers regrowth and functional recovery. As seen in the DRG, after curcumin treatment, the number of sciatic nerve fiber increased [66,69,74,80] in rodent models of sciatic nerve transection, nerve crush injury, and even complete sciatic nerve amputation. The sciatic functional index (SFI) also improved in the above models [66,69,76]. NGF, particularly mature NGF, has also been implicated in regenerative pathways. NGF binds to tropomyosin receptor kinase A (TrkA) and p57 receptors, with mature NGF binding to the former and pro NGF to the latter [78]. As such, TrkA is involved in protective and regenerative pathways while p57 is involved in apoptosis [78]. Downstream of TrkA is the activation of phosphoinositide 3-kinase (PI3K)/Akt pathway, which inhibits mitochondrial damage and thus cell death [78]. In both in vitro (PC12 cell line) and in vivo studies, curcumin increased TrkA, Akt, and mature NGF levels while decreasing pro NGF and caspase 3 levels, resulting in apoptotic reduction. Curcumin therefore has the capacity to promote remyelination and regeneration through modulation of the NF-κB, JNK and PI3K/Akt pathways. However, more work needs to be conducted to unravel curcumin’s regenerative mechanisms as it is not as well understood as remyelination.

Interestingly, an in vitro study shows that curcumin at low doses stimulates the proliferation, migration and lamellipod formation of SCs [35]. In addition, curcumin have been shown to protect axons from degeneration induced by local neuroinflammation in vitro [36]. In addition, low concentrations of curcumin stimulate the proliferation of embryonic neuronal progenitor cells in vitro [96]. Another in vitro study on neurite outgrowth inhibition in PC12 by cisplatin showed reduction of cisplatin-induced inhibition of neurite outgrowth by up to 50% with curcumin treatment [97]. Finally, curcumin has also been reported in vitro to act as a type II positive allosteric modulator of α7-nicotinic acetylcholine receptors (α7-nAchR), decreasing their desensitization and even promoting reactivation of the desensitized pool in Xenopus oocytes expression human receptors [98]. This translates into decreased nociception (reduced acetic acid-induced stretching and reduced paw licking with plantar formalin injection) in in vivo studies and the same therapeutic mechanism has been postulated for curcumin’s effect on microglia [98].

## 5. Curcumin in Clinical Studies for PN

Because of the abundant amount of preclinical data showing the antioxidant and anti-inflammatory properties of curcuminoids, most clinical studies using curcumin have investigated its therapeutic effects in patient diagnosed with chronic inflammatory joint pain, such as osteoarthritis and rheumatoid arthritis. In many of the most recent randomized clinical trials, patients received various formulations of oral curcumin which were designed to enhance its bioavailability compared to the traditional powered extract, such as BCM-95 ^®^, Theracurmin ^®^ and Meriva ^®^. BCM-95 ^®^ and Theracurmin^®^ were shown to reduce knee pain scores respectively in patients with rheumatoid arthritis [99] and osteoarthritis [100]. Meriva^®^ was able to reduce knee pain and blood plasma levels of the inflammatory markers IL-β1 and IL-6 in patients with osteoarthritis [101].

However, clinical studies showing curcumin efficacy in patients diagnosed with PN are substantially lacking. Di Piero et al. (2013) [102] studied the impact of curcumin on chronic neuropathic pain in patients diagnosed with lumbar disc herniation and/or lumbar canal stenosis or carpal tunnel syndrome. Patients were segregated into one of three groups where they received Seractil (dexibuprofen 400 mg/tablet, twice/day), Seractil plus Tiobec 400 (lipoic acid 400 mg/tablet, twice/day), or Seractil plus Lipicur (400 mg lipoic acid with 400 mg curcumin and 4 mg piperine) for 8 weeks. The addition of curcumin to dexibuprofen and lipoic acid regimen significantly reduced neuropathic pain scores in both carpal tunnel and lumbar sciatica patients at 8 weeks post intervention. Curcumin was also shown to reduce the use of dexibuprofen by almost 3 weeks in these patients. Although these results seem promising for the therapeutic use of curcumin in patients with chronic neuropathy, it is impossible to distinguish if these beneficial effects are the result of the bioactive properties of curcumin or coactivity with lipoic acid.

In another observational study by Belcaro et al. 2013 [103], 80 cancer patients undergoing chemotherapy treatment orally received either one tablet of Meriva^®^ 500 mg/day or a placebo for four months. Patients in the Meriva ^®^ group self-reported significantly lower incidence of side effects from chemotherapy, which was further confirmed via semi-quantitative evaluation of cancer chemotherapy side effects, compared to those in the control group. Additionally, plasma free radical levels at the end of the study were observed to be reduced from levels at inclusion in patients given Meriva, while free radical levels increased in the control group.

Asadi et al. 2019 [104] led an 8-week double-blind randomized clinical trial that enrolled 80 patients with type 2 diabetes mellitus. Patients were diagnosed with diabetic peripheral neuropathy via Toronto Clinical Neuropathy Score (TCNS), with a blinded neurologist performing the clinical assessments before and after treatment. Patients were segregated into two groups which received either a polysorbate 80 placebo or an 80 mg nano-curcumin supplement composed of 72% curcumin, 25% desmethoxycurcumin, and 3% bisdemethoxycurcumin. Patients in the curcumin group self-reported a significant decrease in the mean scores of depression and anxiety, assessed via DASS-21-items questionnaire, 8 weeks post treatment. However, when the clinicians compared patient TCNS results, they did not observe any significant differences in the severity of neuropathy at inclusion or 8 weeks post treatment between the curcumin and placebo groups.

A case study by Burns et al. 2009 [105] focused on a 15-year-old, Caucasian, female diagnosed with Déjérine-Sottas disease, a hereditary neurological disorder characterized by damage to the peripheral nerves and resulting in progressive muscle wasting, who was given non-formulated, powdered curcumin in capsules for 12 months as a potential therapeutic regimen for her PN. The patient orally administered 50 mg/kg/day of curcumin (6 × 250 mg capsules three times/day, 1500 mg total) for the first 4 months, and then 75 mg/kg/day (10 × 250 mg capsules three times/day, 2500 mg total) for the remaining 8 months of this 12-month study. After the treatment, the patient displayed no adverse effects but little or no improvement in outcome measures such as muscle strength and upper/lower extremity disability. Similarly, the patient’s neurophysiologic findings were unchanged after 12 months of curcumin treatment. The authors suggested that the patient may not have improved because of her neuropathy possibly having progressed to a point where the potential for recovery was limited, the efficacy of curcumin in patients with Déjérine-Sottas disease may be mutation-dependent, doses used may have been inadequate, or that the dose period may have been too short.

As described above, there is promising data to show that curcumin may have merit in alleviating some aspects of neuropathy in various patient populations, however, more clinical studies are needed in order to fully discern the safety and efficacy of curcumin administration in patients diagnosed with PN. Clinical studies using curcumin and its various formulations have shown that these drugs demonstrate a safety profile and have efficacious effects when used to treat patients diagnosed with chronic inflammatory joint pains. Finally, curcumin have anti-tumor effect and so would be a good drug candidate for CIPN treatment (no concerns by oncologists or patients about adverse effects and tumor growth) [106].

## 6. Conclusions

Curcumin is a lipophilic molecule that is rapidly metabolized, leading to low bioavailability. Thus, several studies propose the use of new formulations of curcumin such as emulsions or nanoparticles to improve systemic bioavailability. These approaches are very interesting in the context of several PN in which nerves are affected (diabetes, alcohol, chemotherapy, genetic mutations...). Moreover, these approaches allow to decrease and better control the dose of curcumin used, although it has no proven toxicity. In this respect, there is growing evidence that curcumin nanoparticles have a better effect on oxidative stress than conventional curcumin. Other authors propose in the case of localized lesions, as in the case of traumatic lesion, transection or local inflammation, the use of biofunctionalized conduit with curcumin or local administration at the site of the lesion. Thus, the use of these new approaches (nanoparticles, tubes...) allows a progressive diffusion of curcumin in the target organism or organ. This makes it possible to avoid the “one shot” effect produced by a conventional injection. This diffusion is all the more interesting as it allows a better reduction of inflammation and oxidative stress, which are processes that last over time and can be chronic. Thus, curcumin because of its anti-inflammatory, antioxidant, anti-ER-stress, and neuro-protective properties is the perfect candidate for the treatment of PN. In addition, its lipophilic nature allows it to be integrated into the myelin sheath, thus exerting a powerful antioxidant effect. The role of curcumin on the ER is not very clear at the moment. In our opinion, it represents a major challenge for the future. Indeed, a growing number of studies show the key role of this organelle in the development of numerous PN (diabetes, alcohol, chemotherapy, genetic mutations...). Thus, because of its numerous biological properties, curcumin reduces tissue damage or improves tissue repair (nerve, muscle, DRG) in many PN. These effects are also reflected at the behavioral level by reducing the signs of pain, improving sensory and motor recovery. However, despite a large number of pre-clinical studies (both in vitro and in vivo) on the subject and a large amount of laboratory evidence of curcumin’s efficacy, studies in humans are sorely lacking. Indeed, to date, only four clinical studies have been specifically conducted in humans in the field of PN. These studies, although encouraging, are sometimes carried out on too few subjects and therefore make it difficult to objectify the use of curcumin clinically for PN. However, the large number of studies on inflammatory pain represents hope for the future use of this molecule for the treatment of neuropathies. We therefore believe that the advent of new curcumin formulations represents a key milestone in the treatment of PN in humans.

## Figures and Tables

**Table 1:** Summary of studies.

Experimental Model	Species	Delivery Method	Formulation	Dose (mg/kg/day)	References
Sciatic nerve crush	SD Rat	local (osmotic pumps), 28 days	Curcumin	0.2	[34]
Oxaliplatin-induced neuropathies	SD Rat	oral gavage, 28 days	Curcumin	12.5, 25, and 50	[39]
Oxaliplatin- and cisplatin-induced neuropathies	Wistar Rat	i.p., 32 days	Curcumin	10	[40]
Cisplatin-induced neuropathy	Wistar Rat	oral, 35 days	Curcumin	200	[41]
Vincristine-induced neuropathy	Swiss Mouse	oral, 14 days	Curcumin	30 to 60	[42]
Vincristine-induced neuropathy	Wistar Rat	oral, 14 days	Tetrahydrocurcumin	40 and 80	[43]
Diabetic peripheral neuropathy	SD Rat	i.p., acute and chronic (days 7 to 21)	Curcumin	50	[44]
Diabetic peripheral neuropathy	Wistar Rat	oral, 6 weeks	Curcumin	50 or 100	[45]
Diabetic peripheral neuropathy	SD Rat	i.p., 14 days	Curcumin	200	[46]
Diabetic peripheral neuropathy	SD Rat	oral, 35 days	Curcumin	100	[47]
Diabetic peripheral neuropathy	SD Rat	oral, 28 days	Curcumin	60	[48]
Diabetic peripheral neuropathy	Laka Mouse	oral, 28 days	Curcumin	15 to 60	[49]
Diabetic peripheral neuropathy	SPF Rat	oral, 5 days	Curcumin derivative J147	10 to 100 µM	[50]
Diabetic peripheral neuropathy	SD Rat	oral, 14 days	Nano-emulsified curcumin	30 to 300	[51]
Diabetic peripheral neuropathy	SD Rat	i.v., 2 injections (week 7 and 8)	Nanoparticle-encapsulated curcumin	16	[52]
Sciatic nerve chronic constriction injury	SD Rat	i.p., 7 days	Curcumin	20, 40 and 60	[53]
Postoperative pain (surgical paw incision)	SD Rat	p.o., acute	Curcumin	10 to 40	[54]
Sciatic nerve chronic constriction injury	SD Rat	oral, 7 days	Curcumin	50	[55]
Sciatic nerve chronic constriction injury	Wistar Rat	i.p., 1 week	Curcumin	12.5, 25, and 50	[56]
Sciatic nerve chronic constriction injury	C57BL/6J Mice	p.o., 3 weeks	Curcumin	5, 15 or 45	[57]
Spinal nerve ligation	Wistar Rat	Intrathecal and p.o.	Curcumin	i.t. 30 to 300 μg / p.o. 10 to 310	[58]
Sciatic nerve section	BALB/c Mouse	i.p., twice daily for 7 days	Curcumin	30 to 120 mg/kg	[59]
Brachial plexus avulsion	SD Rat	i.p., 28 days	Curcumin	60	[60]
Alcohol-induced neuropathy	Wistar Rat	oral, 70 days	Curcumin	20 to 80	[61]
Alcohol-induced neuropathy	Wistar Rat	i.p., 63 days	Curcumin	60	[62]
Opioid-induced hyperalgesia	C57BL/6J Mice	i.p., 6 days	Curcumin	50	[63]
HIV-gp120-induced neuropathic pain	SD Rat	i.v., 3 injections (days 7, 10 and 13)	Nanoparticle-encapsulated curcumin	4	[64]
Complete Freund’s adjuvant induced neuropathic pain	Charles-Foster Rat	i.p., acute	Curcumin	100	[65]
Sciatic nerve crush in diabetic condition	SD Rat	i.p., 28 days	Curcumin	50 to 300	[66]
Diabetic peripheral neuropathy	Swiss Mouse	oral gavage, twice daily 20 weeks	Curcumin derivative J147	10 to 50	[67]
Sciatic nerve crush	Wistar Rat	oral, 28 days	Curcumin	100	[68]
Sciatic nerve excision	Wistar Rat	local (nerve conducts)	Curcumin	10 µL at 5 mg/mL	[69]
Hereditary peripheral neuropahty (CMT1A)	Mouse (*Tr-J*)	oral, 90 days	Curcumin	100	[70]
Hereditary peripheral neuropahty (CMT1A)	Mouse (*Tr-J*)	oral, 90 days	Curcumin	100	[71]
Hereditary peripheral neuropahty (CMT1A)	Rat SD *(PMP22)*	i.p., 8 weeks	Curcumin–cyclodextrin/cellulose Nanocrystals	0.2	[72]
Hereditary peripheral neuropahty (CMT1B)	Mouse (R98C)	oral, 39 days	Curcumin	100	[73]
Sciatic nerve amputation	BALB/c Mouse	oral, 7 days	Curcumin	20 to 40	[74]
Sciatic nerve chronic constriction injury	SD Rat	i.p., 14 days	Curcumin	100	[75]
Sciatic nerve crush	SD Rat	oral, 28 days	Curcumin	100	[76]
Sciatic nerve crush	Wistar Rat	i.p., 4 weeks	Curcumin	100	[77]
Sciatic nerve crush	SD Rat	i.p., 28 days	Curcumin	100	[78]
Sciatic nerve crush	SD Rat	i.p., 60 days	Curcumin	100	[79]
Sciatic nerve transection	Wistar Rat	i.p., 28 days	Curcumin	100	[80]
